# Commentary: Cancer in connective tissue disease

**DOI:** 10.3389/fimmu.2025.1641619

**Published:** 2025-07-22

**Authors:** Jiayi Chen

**Affiliations:** Department of Stomatology, Suzhou Wujiang District Hospital of Traditional Chinese Medicine, Suzhou, China

**Keywords:** autoantibodies, autoimmunity, connective tissue disease (CTD), immunology, malignancy

I read with great interest the comprehensive review by Tonutti et al. titled “Cancer in Connective Tissue Disease” (1), which provides a timely analysis of the bidirectional relationship between malignancy and autoimmunity in connective tissue diseases (CTDs). The authors adeptly synthesize current evidence on cancer risk stratification, autoantibody profiles, and screening challenges across systemic lupus erythematosus, systemic sclerosis, idiopathic inflammatory myopathies (IIM), and Sjögren’s syndrome (SS). Their work underscores the critical need for multidisciplinary collaboration to address unmet needs in early detection and management.

I commend the authors for highlighting the paradoxical role of autoimmunity—where chronic inflammation may promote oncogenesis, yet autoimmune responses can also exert antitumor effects. This duality is exemplified by the contrasting implications of autoantibodies like anti-TIF1-γ (high cancer risk in IIM) and anti-Sp4/CCAR1 (potentially protective). However, I emphasize the urgent need for standardized autoantibody detection methods. As noted, discrepancies in anti-NXP2 results across assays (e.g., line blot vs. immunoprecipitation) complicate clinical interpretation (2). Harmonizing laboratory techniques is essential to refine risk stratification and validate guidelines like the IMACS cancer-screening algorithm (3).

I also support the call for disease-specific screening frameworks. While IMACS offers a model for IIM, similar protocols are lacking for systemic sclerosis and Sjögren’s syndrome, where lymphoma risk escalates with biomarkers like ectopic germinal centers or CXCL13. Tailored strategies must integrate serological, clinical, and imaging data (e.g., salivary gland ultrasound in SS) while balancing cost-effectiveness and accessibility.

Finally, the impact of immunosuppressants on cancer risk warrants deeper exploration. Although the review notes inconclusive data on therapies like mycophenolate in systemic sclerosis, real-world studies are needed to clarify risks associated with newer biologics (e.g., rituximab) and the potential protective role of hydroxychloroquine. Pharmacovigilance registries could illuminate these associations.

In conclusion, Tonutti et al. have delivered an invaluable review that crystallizes the complex cancer-CTD interplay. Future efforts should prioritize validating autoantibody panels, expanding screening guidelines, and elucidating treatment-related oncogenic risks through international cohorts.
